# MENTAL HEALTH AND WELLBEING ACROSS THE COVID-19 PANDEMIC AMONG OLDER PEOPLE IN ENGLAND

**DOI:** 10.1093/geroni/igad104.1377

**Published:** 2023-12-21

**Authors:** Brian Beach, Paola Zaninotto

**Affiliations:** University College London, London, England, United Kingdom; University College London, London, England, United Kingdom

## Abstract

Numerous studies have identified declines in mental health and psychosocial wellbeing over the course of the COVID-19 pandemic across the world and in different age groups, including older people. A key question for researchers, health practitioners, and policymakers alike is whether the influence of the pandemic on these outcomes is temporary or more long-lasting. The lived experience of the pandemic, such as lockdowns, likely played a role in shaping mental health and wellbeing, but recovery is not always immediate following the removal of such negative stimuli. Our recent work using the English Longitudinal Study of Ageing (ELSA) identified that depression, anxiety, and quality of life demonstrated statistically significant worsening trajectories between ELSA Wave 9 (2018/19) and through two assessments during the pandemic, i.e. June/July and Nov/Dec 2020. Because depression and anxiety are also common neuropsychiatric symptoms among people with dementia or mild cognitive impairment, we also examined differences according to cognitive function, finding a convergence among cognitive function groups in the risk for poor mental health through the pandemic. This paper expands on this work to incorporate ELSA Wave 10, the first survey assessment after the easing of key pandemic-related restrictions and health crisis measures, to assess how older people are faring since the acute phase of the pandemic in terms of mental health and wellbeing. It highlights key subgroup dynamics, including cognitive function group, that are associated with patterns of recovery in mental health and wellbeing to pre-pandemic levels and those linked to more long-lasting adverse experiences.

